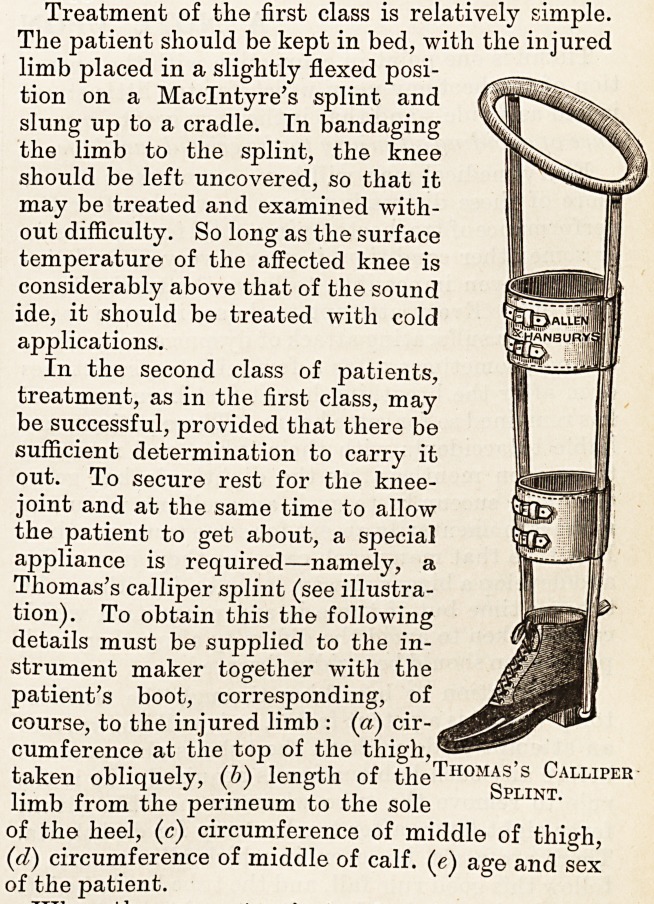# Traumatic Synovitis of the Knee

**Published:** 1907-06-15

**Authors:** 


### Points in Surgery.

### TRAUMATIC SYNOVITIS OF THE KNEE.

### II. TREATMENT.

There are two fundamental principles in the treatment of traumatic synovitis of the knee. The first is to overcome any locking that may be present, and the second is to procure rest for the injured joint.

#### Reduction of Locking.

By locking of the knee is meant inability to extend the leg completely on account of the interposition of a displaced fragment of cartilage or a loose body between the ends of the femur and tibia. Until reduction has been brought about, either by chance or by definite procedure, recovery is not to be expected. Yet locking is sometimes overlooked; and it may be thought to exist when it is absent. If an effusion into the synovial cavity be very copious, so that the joint capsule is tense and hard, the patient will naturally have his knee flexed, because the capacity of the joint is greatest with this posture; and in such a case complete extension of the leg may be impossible. But this limitation can be distinguished from true locking by palpation of the knee while the effort to extend the leg is being made. The sudden check due to true locking is in this way differentiated from the gradual limitation due to distension of an inflamed capsule. Moreover, the patient will be able to judge from his own sensations whether the limitation of extension is or is not due to the interposition of something in the joint. An accurate history of the injury also will aid in the diagnosis, for, if true locking be present, the patient will have been unable to straighten the limb immediately after the injury was received and before the swelling of the knee appeared.

Reduction is performed most satisfactorily with the patient anæsthetised, both because the process is apt otherwise to be painful and because it is easier to make sure that the reduction is complete.

The manipulation required is to flex the leg on the thigh to the fullest degree possible, to pull upon the tibia so as to separate the articular surfaces, to rotate the leg outwards and inwards, and then to extend. If reduction be not effected at the first attempt the manœuvre should be repeated with varying amounts of flexion and rotation.

#### How to Secure Rest.

The proper treatment of an injured knee, like that of any other joint, usually involves complete rest, continued until swelling, increase of temperature, and all other signs of inflammation have disappeared, and the patient can move the joint without pain. By complete rest we must be understood to mean freedom from all adventitious movements. For the surgeon may decide to move the injured joint from time to time, in order to avoid subsequent stiffness due to the formation of adhesions. But organised adhesions, like other forms of scar tissue, take some time to form, and it is best to carry out any gentle movements which may be thought necessary for their prevention under the immediate supervision of the surgeon, perfect immobility being maintained in the intervals. Although this is the ideal to be striven for, the majority of patients have not the money, the time, or the patience necessary for its attainment, so that imperfect measures only are available.

With regard to their ability to secure good treatment patients may be divided into three classes, as follows: (1) Those who can afford to rest at home until the knee is well; (2) those who are obliged to get about, but can afford to pay for an appliance; (3) those who cannot rest and who cannot pay for an appliance.

Treatment of the first class is relatively simple. The patient should be kept in bed, with the injured limb placed in a slightly flexed position on a MacIntyre's splint and slung up to a cradle. In bandaging the limb to the splint, the knee should be left uncovered, so that it may be treated and examined without difficulty. So long as the surface temperature of the affected knee is considerably above that of the sound ide, it should be treated with cold applications.

Thomas's Calliper Splint.

In the second class of patients, treatment, as in the first class, may be successful, provided that there be sufficient determination to carry it out. To secure rest for the knee-joint and at the same time to allow the patient to get about, a special appliance is required—namely, a Thomas's calliper splint (see illustration). To obtain this the following details must be supplied to the instrument maker together with the patient's boot, corresponding, of course, to the injured limb: (*a*) circumference at the top of the thigh, taken obliquely, (*b*) length of the limb from the perineum to the sole of the heel, (*c*) circumference of middle of thigh, (*d*) circumference of middle of calf. (*e*) age and sex of the patient.

When the apparatus is fitted and the patient's boot is laced up, he will be able to walk without movement of, or pressure on, the injured joint, the weight of the body being transmitted from the ischial tuberosity and surrounding parts to the splint. The patient should be obliged to walk with the limb slightly abducted. If this be not so the splint is probably too short, in which case it will fail in its purpose.

Treatment of the third class of patients is often unsatisfactory. As they can neither lie up nor obtain a proper appliance, shift has to be made with such imperfect measures as fixing the leg in a plaster-of-Paris casing, or applying a back splint, or merely strapping the knee or using a Scott's dressing. These methods of treatment all suffer from the great defect that with every step he takes the patient's weight is transmitted through the injured joint. On this account recovery is likely to be tedious, slow, and incomplete. For if rest be not secured for an injured joint, osteo-arthritic changes are apt to ensue.

Of the methods mentioned, a good plaster-of-Paris splint is the best. But there are two precautions to be observed in its application. In the first place, it should extend from the ankle to the top of the thigh, otherwise complete immobility will not be obtained; and, secondly, the knee should be in a position of very slight flexion, for fixation of the leg in the fully extended or slightly over-extended position is most irksome. Moulded splints of guttapercha or poroplastic felt commonly suffer from the defect of being too short.

Wooden back-splints are to be condemned. They slip about, thus failing to immobilise the joint, they are clumsy and uncomfortable. Iron malleable splints are better, but are not so good as plaster of Paris. If back splints be used at all they should be well padded, especially at the level of the knee.

Strapping may prevent stretching of the capsule of the joint by copious effusion, but beyond this it is hardly to be considered as serious treatment.

Operative measures rarely will be required, except in cases of recurrent synovitis, a subject which we hope to discuss in a future article on internal derangement of the knee-joint.

In conclusion, it may be said that the treatment of any case of pure traumatic inflammation of the knee must be persistent and determined, in order to prevent permanent damage to the joint.

## Figures and Tables

**Figure f1:**